# Avoiding alkaline taste through ionotropic receptors

**DOI:** 10.1016/j.isci.2024.110087

**Published:** 2024-05-23

**Authors:** Prakash Pandey, Bhanu Shrestha, Youngseok Lee

**Affiliations:** 1 Department of Bio & Fermentation Convergence Technology, Kookmin University, Seoul 02707, Republic of Korea

**Keywords:** molecular biology, neuroscience

## Abstract

Taste organs contain distinct gustatory receptors that help organisms differentiate between nourishing and potentially harmful foods. The detection of high pH levels plays a crucial role in food selection, but the specific gustatory receptors responsible for perceiving elevated pH in foods have remained unknown. By using *Drosophila melanogaster* as a model organism, we have uncovered the involvement of ionotropic receptors (IRs) in avoiding high-pH foods. Our study involved a combination of behavioral tests and electrophysiological analyses, which led to the identification of six *Irs* from bitter-sensing gustatory receptor neurons essential for rejecting food items with elevated pH levels. Using the same methodology, our study reevaluated the significance of Alka and OtopLa. The findings highlight that Alka, in conjunction with IRs, is crucial for detecting alkaline substances, whereas OtopLa does not contribute to this process. Overall, our study offers valuable insights into the intricate mechanisms governing taste perception in organisms.

## Highlights

• Bitter-sensing gustatory receptor neurons detect alkaline substances
• IRs, but not GRs, are key in sensing alkaline compounds
• IR20a, IR47a, IR51b, IR52a, IR92a, and IR94f are essential for high pH perception
• IR25a and IR76b are not required for detecting alkali

## Graphical abstract

**Figure:**
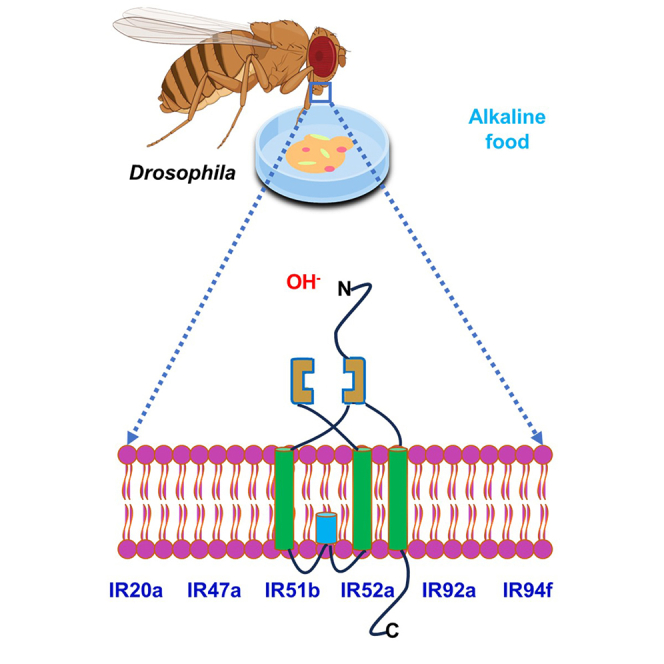

## Introduction

The survival and well-being of animals hinge significantly on their sensory perception, allowing them to navigate their surroundings and fulfill essential needs like obtaining food, locating shelter, achieving reproductive success, ensuring safety, and participating in meaningful interactions with other members of their ecosystem. Among these sensory organs, the proboscis tip comprises the bifurcate labellum, which plays a crucial role in taste detection by contacting food or chemical compounds. In *Drosophila*, the labellum is equipped with 31 taste sensilla on each side, arranged in a stereotypical pattern. These sensilla are pivotal in chemosensations, particularly for non-volatile compounds. The labellum consists of three distinct sensilla types: short (S-type), intermediate (I-type), and long (L-type), distinguished by their sizes.^1^^,^^2^^,^^3^ Each sensillum receives innervation from two to four gustatory receptor neurons (GRNs), one mechanosensory neuron, and three supporting cells. The signals from these cells are then projected to the subesophageal zone of the brain, responsible for taste perception. The GRNs within the sensilla exhibit sensitivity to various chemical stimuli that enter through pores at the sensilla’s tip. Regarding S-type sensilla, four distinct GRNs are present, each responsive to bitter or aversive compounds, sweet tastes, water, and specific minerals like Na^+^ and Ca^2+^.^4^^,^^5^^,^^6^ In contrast, the L-type sensilla feature four sets of GRNs that exhibit sensitivity to sweet tastes, water, low salt concentrations, and other compounds that remain unidentified.^1^^,^^7^^,^^8^ Likewise, the I-type sensilla are outfitted with two sets of GRNs that respond to both sweet and bitter compounds. Hence, flies can detect a range of tastes, including sweet, bitter, salty, sour, and certain amino acids.^9^^,^^10^^,^^11^^,^^12^^,^^13^^,^^14^^,^^15^^,^^16^

Sourness, defined by low pH or acidity, constitutes a fundamental taste sensation.^17^ It is universally attractive in moderate concentrations but becomes unappealing at higher levels.^18^ In contrast, foods with high pH or alkaline properties typically lack appeal. Acidic components are usually present in raw fruits and spoiled food items, underscoring the importance of detecting sourness as a warning sign.^19^ On the contrary, basic or alkaline taste is elicited in certain vegetables, legumes, and other items due to their elevated pH levels. Recent research across diverse species has confirmed that basic taste also qualifies as one of the fundamental taste qualities.^20^^,^^21^^,^^22^^,^^23^

Research conducted on carabid beetles and ground beetles has revealed a robust aversion to alkaline conditions, particularly concerning their habitats and food sources.^24^ These studies have proposed the existence of taste receptors in insects capable of detecting elevated pH levels, which influences their feeding behavior. This investigation highlights the ecological significance of an aversion to alkaline conditions, which influences habitat selection, soil quality, vegetation composition, and community dynamics, ultimately affecting conservation and land management practices, as well as ecosystem diversity and productivity, by favoring species adapted to neutral or slightly acidic soils, limiting nutrient availability, promoting alkaline-tolerant vegetation, and altering species interactions and succession patterns across diverse ecosystems. This underscores the intricate network of interactions and dependencies characterizing ecological systems. In the context of *Drosophila* research, it was observed that alkaline substances with high pH can indeed elicit a gustatory sensation, indicating the presence of a distinct channel in the taste organ dedicated to alkaline taste perception. Subsequent exploration uncovered the pivotal role of a chloride channel named alkaliphile (Alka) in fruit flies’ aversive taste responses to basic foods.^20^ Alka selectively forms a high pH-gated chloride channel in specific GRNs, enabling the detection of alkaline taste. Furthermore, investigations in vertebrates, particularly zebrafish, have unveiled the role of otopetrin-1 (OTOP1) in basic taste sensation.^21^ OTOP1 was identified as a mediator of proton inflow and efflux in response to extracellular acid and base stimulation. Significantly, the mutation of specific domains within OTOP1 was observed to alter its affinity for alkali compounds, without affecting its response to acidic stimuli, underscoring the differentiation between acid and alkali activation.

A crucial aspect of this discrimination lies in the identification of high pH levels, which, until now, has remained enigmatic in terms of the specific gustatory receptors (GRs) involved. Employing *Drosophila melanogaster* as our model organism, we initiated an exhaustive investigation that unveiled the pivotal role of ionotropic receptors (IRs) in helping flies avoid high-pH foods. Potential candidates such as Alka and OtopLa, an ortholog of mammalian OTOP1, were simultaneously explored. Our research comprised a combination of behavioral assessments and electrophysiological analyses, ultimately leading to the identification of six IRs originating from bitter-sensing GRNs. These six IRs were determined to be indispensable for flies in rejecting food items characterized by elevated pH levels. In essence, our study highlights the distinctive function of IRs in insects when concerning the detection of foods with high pH content.

## Results

### Bitter GRNs detect alkaline substances

**Figure 1:**
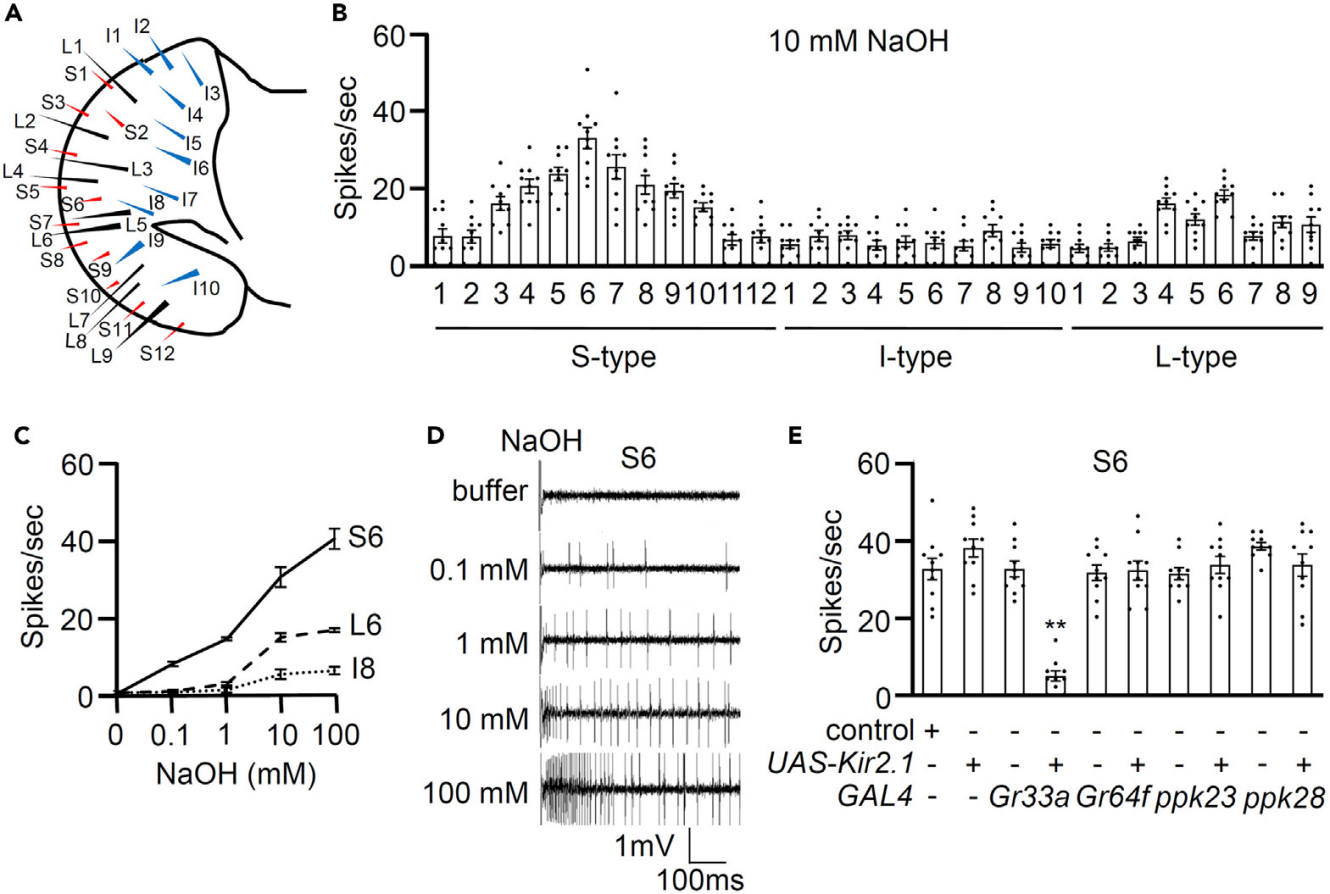
Bitter gustatory receptor neurons detect alkaline substances (A) Diagrammatic representation of the gustatory sensilla on the fly labellum. (B) Neurophysiological mapping was conducted on all S-type, I-type, and L-type labellar sensilla in the presence of 10 mM NaOH using control (*w*^*1118*^) flies (*n* = 10). (C) Electrophysiological analysis was conducted with different NaOH doses ranging from 0.1 to 100 mM from S6, I8, and L6 sensilla (*n* = 10). (D) Representative sample traces fired by S6 sensilla of the control fly with the buffer and different NaOH doses. (E) Tip recordings were performed in the presence of 10 mM NaOH after inhibiting different gustatory receptor neurons (GRNs) by expressing *UAS*-*Kir2.1* under the control of the indicated *GAL4* drivers on S6 sensilla. +/− indicates the presence or absence of the transgene, respectively (*n* = 10). Data information: All error bars represent the standard error of the mean (SEM). Single-factor analysis of variance (ANOVA), coupled with Scheffe’s post hoc test, was conducted to compare multiple datasets. Asterisks indicate statistical significance compared to the control. ∗∗*p* < 0.01**.**

Our objective was to elucidate the molecular mechanisms underlying alkaline taste perception while focusing on recent advancements in detecting alkali compounds. To manipulate the pH of food, we opted for the potent alkaline substance sodium hydroxide (NaOH), as its complete dissociation into Na^+^ and OH^−^ ions upon dissolution provides a versatile array of basic pH levels. Initially, we conducted tip recording assays on 31 labellum sensilla within the fly’s gustatory system when exposed to 10 mM NaOH (Figures 1A and 1B). Among these sensilla, S6 of the S-type sensilla exhibited the highest responsiveness to NaOH, while minimal neuronal firing was observed in I- and L-type sensilla. Subsequently, we chose S6, L6, and I8 for measuring dose-dependent responses (Figures 1C and 1D). The response of the S6 sensilla to NaOH showed a significant dose-dependent increase. In contrast, the L6 sensilla displayed slightly elevated responses at 10 and 100 mM NaOH, though the differences were not statistically significant. Furthermore, I8 did not show activation within the 0.1 to 100 mM range of NaOH.

Further experiments involved selectively deactivating specific GRN types in flies using the inwardly rectifying potassium channel, *UAS*-*Kir2.1*, under the control of *GAL4* drivers.^25^ This inhibition effectively reduced action potentials in S6 sensilla under the *Gr33a*-*GAL4*/+ background (associated with bitter-sensing GRNs).^26^ However, this inhibition did not affect the action potentials in S6 under the *Gr64f*-*GAL4* (sweet-sensing GRNs), *Ppk23*-*GAL4* (salt-sensing GRNs), and *Ppk28*-*GAL4* (water-sensing GRNs) backgrounds (Figure 1E).^5^^,^^27^^,^^28^^,^^29^

Regarding behavioral avoidance, the role of the bitter-sensing GRN (*Gr33a*-*GAL4*) was also observed as a result of physiology. Recently, Alka has been shown to be expressed in 21.6% of bitter-sensing GRNs (*Gr66a*-*GAL4*).^20^ *Gr33a* is co-expressed with *Gr66a*.^26^ To confirm the importance of bitter-sensing GRNs, we conducted an aversive behavior test. Animals instinctively choose nourishing foods and avoid toxins based on taste preferences. In a binary food choice assay spanning a range of 0–100 mM NaOH (Figure S1A), wild-type flies predominantly avoided the highest NaOH concentration. Consistent with the electrophysiology results, flies ablated of bitter-sensing GRNs, but not the others, had defects in avoiding alkaline substances (Figure S1B). Additionally, the study reveals a puzzling phenomenon: even though only 21.6% of neurons overlap between Alka and bitter-sensing GRNs (*Gr66a*-*GAL4*),^20^ and Alka is specifically attuned to detecting alkaline solutions, it raises a compelling question as to why flies with ablated bitter-sensing GRNs still exhibit deficiencies in avoiding alkaline substances.

### IRs play a crucial role in detecting alkaline substances

**Figure 2:**
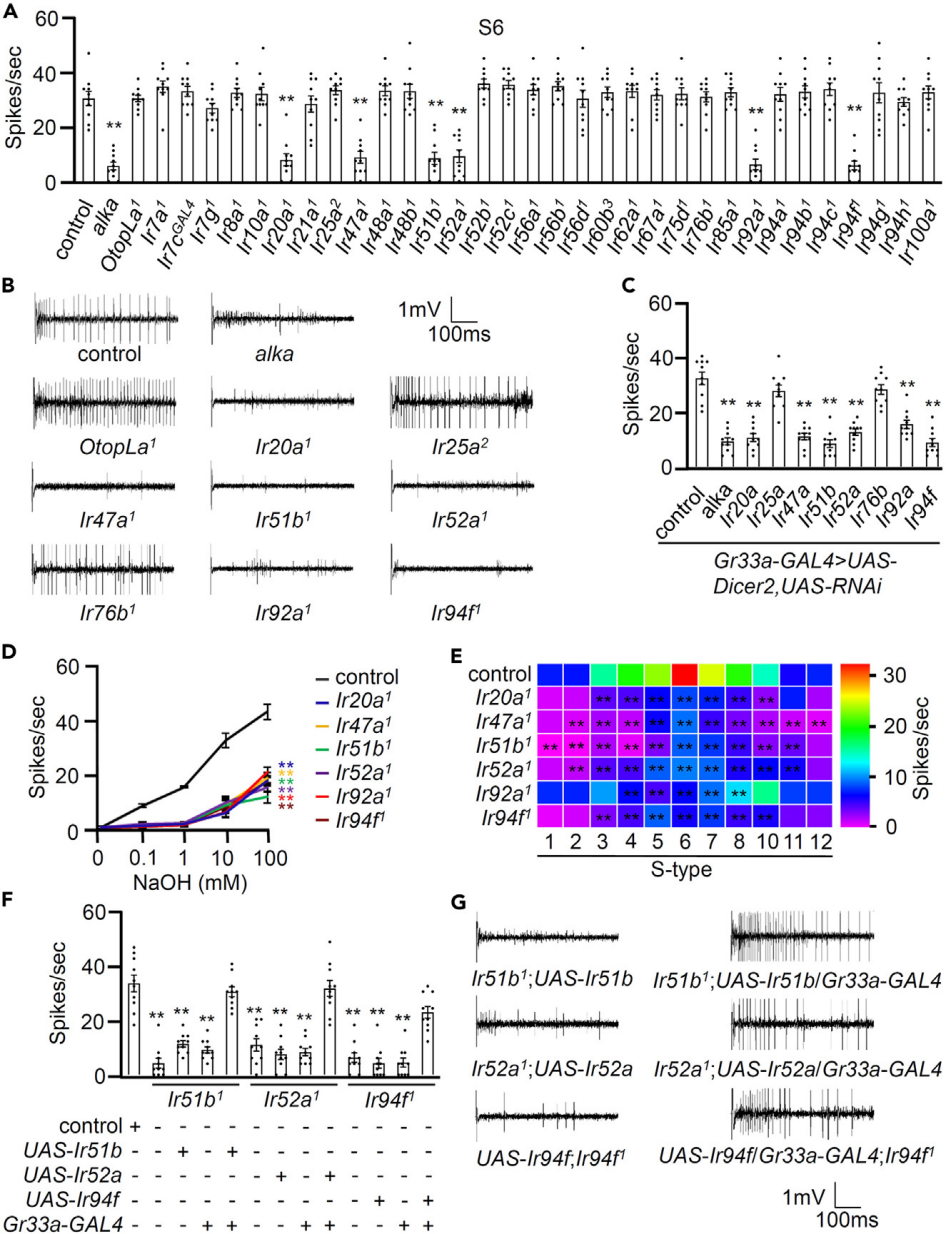
Ionotropic receptors play a crucial role in the detection of alkaline substances (A) Tip-recording analyses were carried out on S6 sensilla in response to 10 mM NaOH for the control, *alka*, *OtopLa*^*1*^, and 31 *Ir* mutants (*n* = 10). (B) Representative sample traces obtained from S6 sensilla of the control, *alka*, *OtopLa*^*1*^, *Ir20a^1^*, *Ir25a^2^*, *Ir47a^1^*, *Ir51b^1^*, *Ir52a^1^*, *Ir76b^1^*, *Ir92a^1^*, and *Ir94f^1^*. (C) Electrophysiological analyses were performed on the control and *Gr33a*-*GAL4*/+;*UAS*-*Dicer2*/*UAS*-RNAi flies using the following lines: *alka*, *Ir20a*, *Ir25a*, *Ir47a*, *Ir51b*, *Ir52a*, *Ir76b*, *Ir92a*, and *Ir94f* RNAi (*n* = 10). (D) Dose-responsive nerve firing was recorded from S6 sensilla at concentrations ranging from 0.1 to 100 mM NaOH for both the control and mutants (*Ir20a*^*1*^, *Ir47a*^*1*^, *Ir51b*^*1*^, *Ir52a*^*1*^, *Ir92a*^*1*^, and *Ir94f*^*1*^) (*n* = 10). (E) Tip-recording analyses were performed on different S-type sensilla of the control and six mutants (*Ir20a^1^*, *Ir47a^1^*, *Ir51b^1^*, *Ir52a^1^*, *Ir92a^1^*, and *Ir94f^1^*) by applying 10 mM NaOH as stimuli (*n* = 10). (F) Rescue of the mutant deficit in the tip recordings from S6 was achieved by driving the respective *UAS* lines with the broadly tuned *Gr33a*-*GAL4* in the bitter-sensing GRNs. +/− indicates the presence or absence of the transgene, respectively (*n* = 10). Nerve firing activity originating from S6 sensilla was recorded at a concentration of 10 mM NaOH. (G) Representative sample traces obtained from S6 sensilla in *UAS* parent strains and rescue flies from (F). Data information: All error bars represent the SEM. Single-factor ANOVA, coupled with Scheffe’s post hoc test, was conducted to compare multiple datasets. Asterisks indicate statistical significance compared to the control. ∗∗*p* < 0.01**.**

Generally, sour taste is detected through the evolutionarily well-conserved OtopLa.^11^^,^^12^ However, the detection of aversive acetic acid relies on the essential role of IR7a.^30^ For attractive carboxylic acids, the involvement of IR25a and IR76b, along with some sweet GRs, is required.^13^^,^^31^ IRs predominantly exhibit expression in olfactory and taste organs.^32^^,^^33^ Following a comprehensive review of the literature, we specifically targeted 30 *Ir* mutants known to be expressed in the labellum and taste sensilla surrounding the legs and wing margins.^34^^,^^35^^,^^36^^,^^37^ Notably, *Ir8a* stands out as it is solely expressed in olfactory organs.^38^ To identify the alkaline taste sensor, we employed tip recording assays on an *Ir* mutant line with either known or unclear physiological functions (Figures 2A and 2B). Initially, we incorporated the *alka* mutant as a negative control, representing deficiencies in neuronal activation when exposed to 10 mM NaOH. However, the recently identified mammalian OTOP1 ortholog, *OtopLa*, demonstrated normal responses to alkaline substances. Most mutant flies exhibited similar neuronal responses to alkaline food as the control flies, but six mutants (*Ir20a*^*1*^, *Ir47a*^*1*^, *Ir51b*^*1*^, *Ir52a*^*1*^, *Ir92a*^*1*^, and *Ir94f*^*1*^) displayed significantly reduced responses to alkaline tastants. For example, IR20a is responsible for detecting combinations of amino acids rather than individual amino acids.^39^^,^^40^ IR47a plays a pivotal role in detecting the presence of the toxic element cadmium.^41^ In addition, IR51b is essential for detecting nitrogenous waste and amino acids such as arginine, lysine, proline, as well as varying concentrations of valine, tryptophan, isoleucine, and leucine.^40^^,^^42^ Furthermore, it plays a crucial role in cantharidin sensation.^43^ The collaboration of IR94f with IR7g, IR25a, IR51b, and IR76b is essential for detecting cantharidin in the labellum.^43^ However, it is worth noting that there have been no expression analyses conducted for IR52a and IR92a in the labellum. Intriguingly, despite their broad involvement in other sensory processes, IR25a and IR76b were not found to be essential for the alkali response. To confirm these findings, we used RNA interference (RNAi) to reduce gene levels including *alka* in the bitter-sensing GRNs, effectively eliminating the neuronal response to alkaline tastants, although *Ir25a* RNAi and *Ir76b* RNAi flies in the same GRNs showed normal neuronal responses (Figure 2C). This result contradicts previous findings; Mi et al. demonstrated that *alka* is not expressed in the bitter-sensing GRNs of the S6 sensillum.^20^ Dose-wise tip-recording analyses revealed a significant decrease in action potentials in the six mutants compared to that of the wild-type controls across different concentrations (0.1–100 mM), except for concentrations greater than 1 mM, where the mutants showed an increase in action potentials. This suggests there may be additional types of alkaline receptors responsible for responses to 10–100 mM NaOH, or Na^+^ sensor may induce neuronal activation (Figure 2D). Next, we further analyzed the six mutants on other S-type sensilla (Figure 2E). The control group demonstrated over 10 spikes per second in the neuronal activity from S3 to S10, while most mutants, except *Ir92a*^*1*^, exhibited a significant reduction in their response to alkali. The response of *Ir92a*^*1*^ was relatively milder, as the mutant showed no defects in S3 and S10 sensilla; this suggests that *Ir92a* has a very specific expression pattern in the labellum. Finally, we restored a complete alkaline neuronal response by expressing the wild-type cDNA of the mutants (*Ir51b*^*1*^, *Ir52a*^*1*^, and *Ir94f*^*1*^) in bitter-sensing GRNs driven by broadly tuned *Gr33a*-*GAL4* in electrophysiology (Figures 2F and 2G). It is worth noting that validation experiments for the *Ir20a*, *Ir47a*, and *Ir92a* genes were not conducted using the *GAL4*/*UAS* system. Nevertheless, our study conclusively demonstrated that RNA knockdown of all six genes led to the specific elimination of alkaline neuronal activation within bitter-sensing GRNs. This underscores the pivotal role of the newly identified six IRs in detecting alkali.

### IR20a, IR47a, IR51b, IR52a, IR92a, and IR94f, in conjunction with Alka, are essential components for the aversion response to alkali

**Figure 3:**
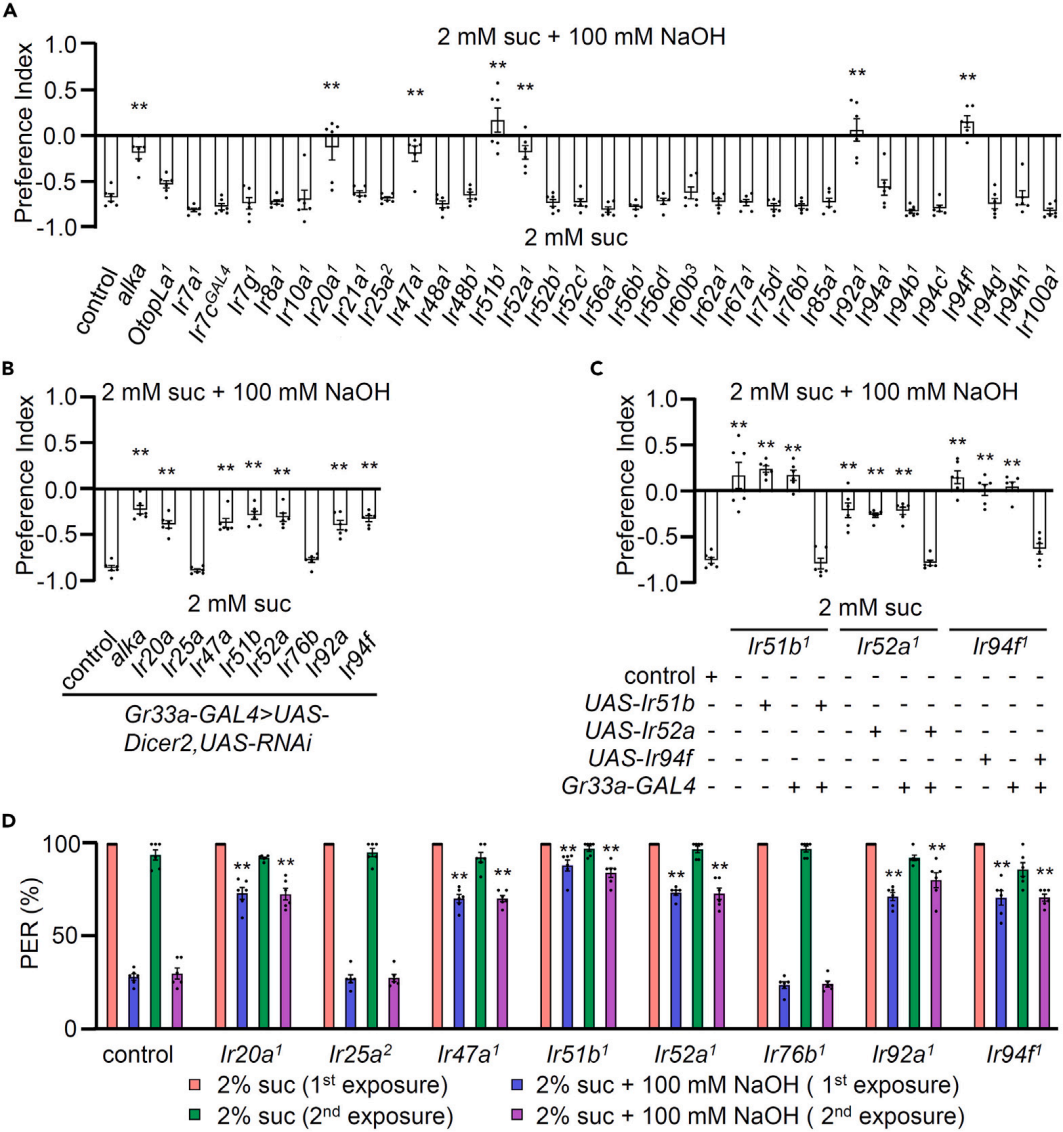
IR20a, IR47a, IR51b, IR52a, IR92a, and IR94f, in conjunction with Alka, are essential components for the aversion response to alkali (A) In the binary food choice assay, 31 *Ir* mutants, *alka*, and *OtopLa*^*1*^ were compared to the control (*n* = 6). (B) The binary food choice assay was performed on the control and *Gr33a*-*GAL4*/+;*UAS*-*Dicer2*/*UAS*-RNAi flies using the following lines: *alka*, *Ir20a*, *Ir25a*, *Ir47a*, *Ir51b*, *Ir52a*, *Ir76b*, *Ir92a*, and *Ir94f* RNAi (*n* = 6). (C) Behavioral rescue of the mutant deficit in the binary food choice assay was accomplished by driving the respective *UAS* lines with *Gr33a*-*GAL4*. +/− indicates the presence or absence of the transgene, respectively (*n* = 6). (D) Proboscis extension response (PER) assay analysis was performed on the control, *Ir20a*^*1*^, *Ir25a*^*2*^, *Ir47a*^*1*^, *Ir51b*^*1*^, *Ir52a*^*1*^, *Ir76b*^*1*^, *Ir92a*^*1*^, and *Ir94f*^*1*^ flies in the presence of 100 mM NaOH (*n* = 6). Data information: All error bars represent the SEM. Single-factor ANOVA, coupled with Scheffe’s post hoc test, was conducted to compare multiple datasets. Asterisks indicate statistical significance compared to the control. ∗∗*p* < 0.01**.**

The integration of physiology with a behavioral paradigm serves as a crucial means of validating research findings. To elucidate the translation of taste receptor activities into behavioral responses, we implemented a binary food choice assay in an unbiased manner. Individual behavioral screenings were performed for all *Ir* mutants and consistently revealed defects in alkali aversion in all cases (Figure 3A). Specific taste receptors, such as IR20a, IR47a, IR51b, IR52a, IR92a, and IR94f, were implicated in this avoidance behavior, while common bitter GRs like GR32a, GR33a, GR66a, and GR89a, as well as the common IR coreceptors, IR25a and IR76b, did not contribute to alkali sensing (Figures 3A and S2). Consistent with the electrophysiology results, *alka*, but not *OtopLa*, was essential for the behavioral assay (Figure 3A). These collective results strongly underscore the crucial role of these six IRs in detecting elevated pH levels. To further validate these behavioral defects, we employed RNA interference driven by *Gr33a*-*GAL4* (Figure 3B) and conducted recovery experiments using the *GAL4*/*UAS* system (Figure 3C). Consistent with the electrophysiology results, these additional analyses reinforced the observed behavioral abnormalities. Additionally, our RNAi data targeting *alka* in the bitter-sensing GRNs strongly suggest that Alka primarily operates within these neurons. However, the defects observed in *alka* mutants should be substantiated through rescue experiments using *Gr33a*-*GAL4* and *UAS*-*alka*.

For a more comprehensive assessment of the behavioral assay, we conducted the proboscis extension response (PER) assay to directly deliver taste stimuli to the labellum (Figure 3D). Initially, we identified flies responsive to sucrose. Subsequently, the aversion response to 100 mM NaOH was assessed twice, with an interval to reevaluate the sucrose response. In this context, the two broadly expressed coreceptors, IR25a and IR76b, exhibited a normal PER response compared to the control. However, the six candidate mutants displayed a significant reduction in alkali avoidance. This compelling evidence supports the assertion that these specific genes play a pivotal role in governing the response to alkali stimuli.

### Six IRs in the labellum are required for sensing high pH

**Figure 4:**
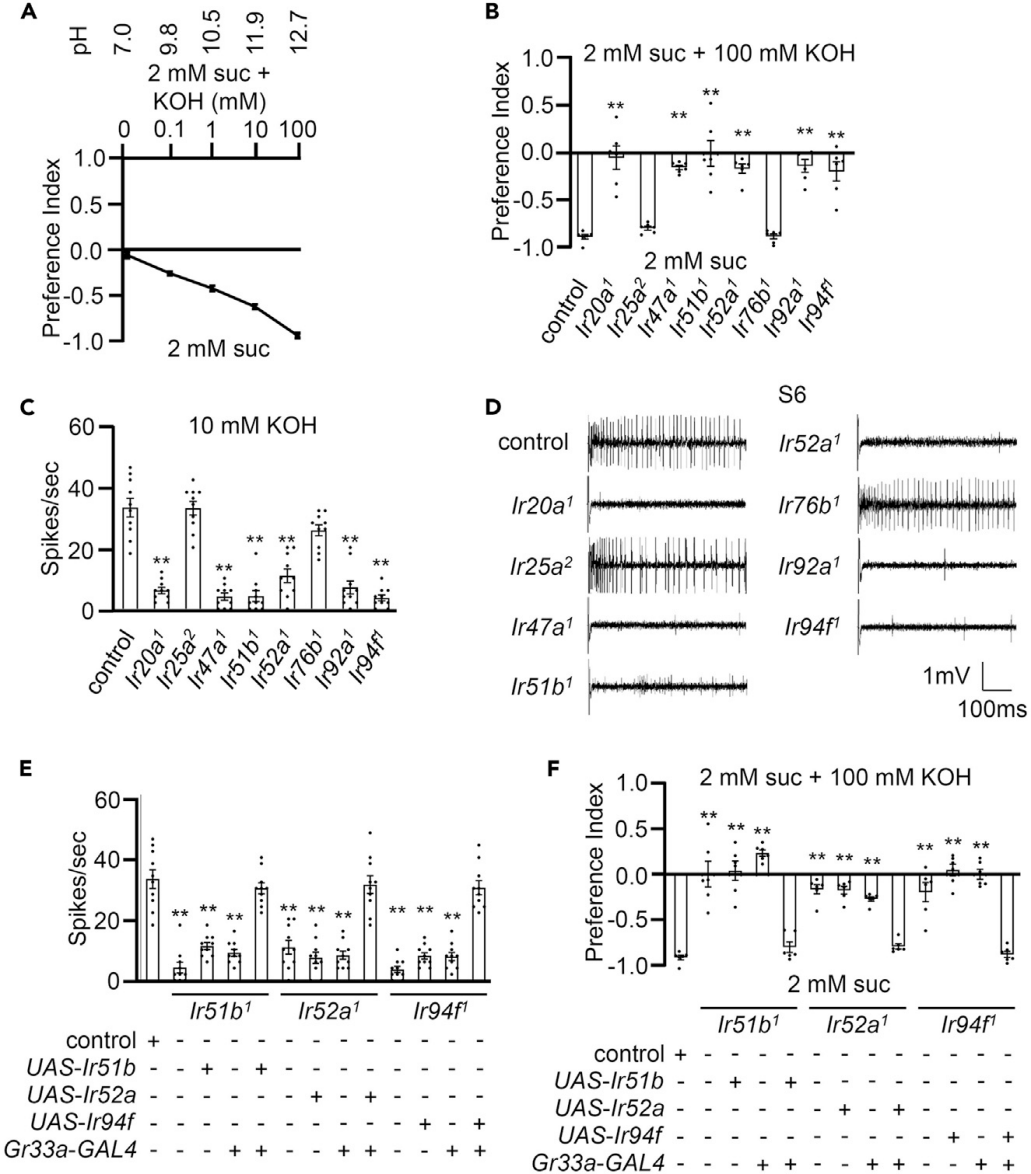
Six IRs in the labellum are required for sensing high pH (A) Dose-dependent binary food choice assays were performed with control flies using KOH in the range of 0–100 mM (*n* = 6). (B) Binary food choice assays were conducted with both the control and mutants (*Ir20a^1^*, *Ir47a^1^*, *Ir51b^1^*, *Ir52a^1^*, *Ir92a^1^*, and *Ir94f^1^*) in the presence of 100 mM KOH (*n* = 6). (C) Electrophysiological analyses were conducted on both the control and mutants (*Ir20a^1^*, *Ir47a^1^*, *Ir51b^1^*, *Ir52a^1^*, *Ir92a^1^*, and *Ir94f^1^*) in the presence of 10 mM KOH (*n* = 10). (D) Representative samples obtained from S6 sensilla of the control, *Ir20a^1^*, *Ir47a^1^*, *Ir51b^1^*, *Ir52a^1^*, *Ir92a^1^*, and *Ir94f^1^* in (C). (E) Rescue of the mutant deficit in the tip recordings was achieved by driving the respective *UAS* lines with the broadly tuned *Gr33a*-*GAL4* in the bitter-sensing GRNs. +/− indicates the presence or absence of the transgene, respectively (*n* = 10). (F) Behavioral rescue of the mutant deficit in the binary food choice assay was accomplished by driving the respective *UAS* lines with *Gr33a*-*GAL4*. +/− indicates the presence or absence of the transgene, respectively (*n* = 6). Data information: All error bars represent the SEM. Single-factor ANOVA, coupled with Scheffe’s post hoc test, was conducted to compare multiple datasets. Asterisks indicate statistical significance compared to the control. ∗∗*p* < 0.01.

Potassium hydroxide (KOH) is acknowledged as a potent base, typically maintaining a pH range between 10 and 13. Significantly, NaOH and KOH share the highest degree of chemical similarity among various hydroxides. During our investigation, we questioned whether the six IRs under scrutiny exhibit specificity toward NaOH or if they play a crucial role in sensing elevated pH levels. To explore this, we conducted a comprehensive dose-wise feeding assay covering concentrations ranging from 0 to 100 mM KOH. The results revealed that, like the NaOH response, *Drosophila* exhibited an aversion toward increasing concentrations of KOH (Figure 4A). This observation suggests a common response to escalating pH levels, implicating the involvement of these receptors in the detection of high pH environments. Furthermore, it is noteworthy that most of our experiments were replicated using KOH instead of NaOH, yielding consistent results in aversion (Figures 4A and 4B), electrophysiology (Figures 4C and 4D), and specificity to the six genes. The mutant defects were also successfully rescued using both electrophysiology and behavioral assays (Figures 4E and 4F). Hence, these findings strongly indicate the specificity of these six IRs in detecting alkaline solutions with high pH.

While we presented functional evidence of each IR in the labellum through electrophysiology and two behavioral assays, the expression of some *Ir*s in the labellum remains unclear. Immunohistochemical examination allowed us to successfully see GFP expression of *Ir47a*-*GAL4* in the labellum; however, we were unable to achieve this result in most of the remaining five reporters for the six *Ir*s. Consequently, we sought to verify whether *Ir47a* is genuinely expressed in the bitter-sensing GRNs. Our investigation then focused on understanding the integration of *Ir47a*^+^ neurons into the broader network of bitter-sensing GRNs in the labellum. For this purpose, we utilized *Gr66a*-*I*-*GFP*, a marker for bitter-sensing GRNs.^44^ Through co-labeling experiments, we revealed a notable coincidence between *Ir47a*^+^ neurons and the repulsive *Gr66a*^+^ neurons (Figure S3A). Interestingly, our findings indicated that the neuronal populations labeled by *Ir47a* and *Gr66a* reporters did not entirely overlap, although a majority did overlap. This result provides compelling evidence that IR47a is indeed associated with bitter GRNs.

Previously, the expression of *Ir51b* and *Ir94f* in the bitter-sensing GRNs was assessed using reverse-transcription polymerase chain reaction (RT-PCR) analyses.^42^^,^^43^ Employing the same method, we subsequently extended our investigation to include *Ir20a*, *Ir52a*, and *Ir92a*, in addition to *Ir51b* and *Ir94f*. To achieve this, we introduced the cell death gene *UAS*-*hid* under the control of the *Gr33a*-*GAL4* receptor, generating labellum samples with ablated bitter-sensing GRNs. Consequently, three types of samples were prepared: wild-type whole body, wild-type labellum, and labellum with ablated bitter-sensing GRNs (Figure S3B). Significantly, the anticipated bands for *Ir51b*, *Ir52a*, and *Ir94f* were successfully identified in the wild-type labella, but these bands disappeared in the labellum samples with ablated bitter-sensing GRNs. However, the expression of *Ir20a* and *Ir92a* in the labellum was not detected using RT-PCR, despite the presence of bands in the samples obtained from the whole body. Consequently, we believe that the expression of *Ir20a* and *Ir92a* in the labellum might be extremely low, falling below the detection threshold of the RT-PCR technique. This comprehensive evidence underscores the pivotal role of IR51b, IR52a, and IR94f in the GRN-mediated perception of aversive compounds.

## Discussion

The concept of alkaline taste plays a crucial role in the intricate tapestry of ecosystems, influencing environmental dynamics and human interactions. Organisms across diverse habitats must maintain a delicate balance, as optimal physiological functions often depend on specific pH levels. Alkaline taste, whether through natural occurrences or anthropogenic influences, significantly impacts the health and behavior of various species. For instance, insect ecology is profoundly influenced by the perception of alkaline taste, shaping feeding preferences, foraging behaviors, and ecological interactions within ecosystems. Hence, understanding and managing alkaline taste becomes essential for sustaining harmonious coexistence with the environment.

In our study, IRs were found to play a role in alkaline taste sensation in *Drosophila*. This contrasts with studies in vertebrates, where OTOP1 is involved in perceiving basic compounds.^21^ Additionally, the role of Alka was previously demonstrated for identifying basic compounds in *Drosophila*.^20^ Alkaline taste has been proposed as a foundational taste alongside sweet, bitter, sour, and salty in animal gustation research. Our findings in *Drosophila*, including a strong aversive reaction to alkaline compounds and the involvement of specific IRs, parallel what has been observed in human taste experiences. The intricate interplay between alkaline taste and the physiology of living organisms underscores the critical role this sensory aspect plays in maintaining equilibrium within diverse biological systems. Living organisms exhibit a remarkable sensitivity to pH levels, with optimal physiological activities occurring within a narrow pH range.^45^ Excessively high pH can disrupt the delicate acid-base balance, potentially leading to life-threatening conditions; therefore, identifying key receptors for alkaline taste sensation becomes pivotal in unraveling the mechanisms underlying these physiological responses. In organisms ranging from simple to complex, the molecular machinery involved in detecting alkaline taste, such as IRs, emerges as a linchpin.

Our observations in *Drosophila* reveal a strong aversive reaction to alkaline compounds, triggering the activation of bitter-sensing GRNs. Notably, the conventional coreceptors, IR25a and IR76b, played no essential role in this mechanism; these coreceptors are well known to function in detecting salt (Na^+^ and Ca^2+^),^7^^,^^8^^,^^15^^,^^46^ amino acids,^40^ carboxylic acids,^13^^,^^31^ ammonia,^42^ metals,^41^^,^^47^^,^^48^ and the bitter compound cantharidin.^43^ However, for the perception of acetic acid, only the role of IR7a was observed, with IR25a and IR76b showing no significant impact on sensing acetic acid.^30^

Specific IRs, including IR20a, IR47a, IR51b, IR52a, IR92a, and IR94f, were identified as crucial in mediating alkaline taste sensing within bitter GRNs. This specificity was confirmed through extensive screening, RNA interference, and recovery experiments. The normal response of IR76b also suggests that the six identified IRs are tuned toward sensing the OH^−^ group rather than Na^+^, as indicated by a recent paper demonstrating their normal response to NaCl.^16^ The distinct aversive response to alkaline food by these identified IRs is attributed to their sensitivity to basic properties.

Our molecular genetics investigation showcases that fruit flies actively avoid highly alkaline food by using their gustatory system. This suggests *Drosophila* as a model organism for exploring alkaline taste sensation. IRs were identified as taste modulators finely tuned to high pH, with IR47a co-existing with bitter GRNs and other IRs (IR51b, IR52a, and IR94f) showing expression in bitter GRNs. Our attempts to detect clear expression of Ir20a and Ir92a in this study were unsuccessful. However, we do not discount the possibility of IR20a and IR92a functioning in the labellum, given that our tip recordings were specific to sensilla in the labellum. Moreover, the combination of tissue-specific ablation through RNA interference and mutant analysis provides substantial evidence supporting the role of IR20a and IR92a in the labellum. Additionally, IR20a has been previously identified as one of the receptors for mixed amino acids.^39^ A limitation of the study lies in our hypothesis regarding the collaborative function of all six IRs, based on the prevalent defects observed in the sensilla. For example, IR25a and IR93a serve as common components in distinct IR pathways, playing critical roles in both thermosensation and hygrosensation.^49^^,^^50^^,^^51^^,^^52^ Similarly, IR51b exhibits multifaceted functionality, being implicated in the detection of nitrogenous wastes, amino acids, and cantharidin within the bitter-sensing GRNs.^40^^,^^42^^,^^43^ Furthermore, our tip recording analysis reveals a relatively wider requirement for IR51b on S-type sensilla (Figure 2E). Consequently, it is plausible that IR51b may serve as a potential co-receptor in the bitter-sensing GRNs. Conversely, the defects observed in *Ir92a*^*1*^ appear comparatively weaker than those of other *Irs*. For instance, *Ir92a*^*1*^ displays normal responses on S10 in electrophysiology, suggesting its non-essential role, at least in this context. We propose a model wherein five or six IRs form a composite structure to collectively detect alkali substances. However, this model requires validation through *in vitro* functional experiments in future research endeavors.

In conclusion, this underscores the involvement of IRs in avoiding alkaline taste in the labellum. The current study establishes 6 IRs as bona fide taste receptors, but not all IRs in flies, if any, dedicated to sensing the basicity of food in *Drosophila*. This provides insights into the mechanisms underlying the fly’s avoidance of alkaline food and enhancing our understanding of taste perception. Our study introduces a crucial role of IRs as insect-specific taste receptors detecting for alkaline foods. This discovery enhances our comprehension of the function of IRs, their diverse roles, and the mechanisms underlying their operation, thus deepening our understanding of insect biology and paving the way for exploring the neural mechanisms responsible for alkaline taste perception across species.

### Limitations of the study

The constraints of this study involve illustrating the co-receptors responsible for the 6 IRs implicated in alkaline taste. The current findings require validation through *in vitro* functional experiments in future research endeavors.

## STAR★Methods

### Key resources table

**Table:** 

REAGENT or RESOURCE	SOURCE	IDENTIFIER
**Antibodies**		

Mouse anti-GFP	Molecular probes	Cat # A11120; RPID: AB_221568
Goat Anti-mouse Alexa Fluro 488	Invitrogen	Cat # A32723; RRID: AB_2633275
Rabbit anti-DsRed	TaKaRa	Cat #632496; RRID: AB_10013483
Goat anti-rabbit Alexa Fluor 568	Thermo Fisher/Invitrogen	Cat #A11011; RRID: AB_143157

**Chemicals, peptides, and recombinant proteins**		

Sodium hydroxide	Sigma-Alderich Co	Cat#S8045
Potassium hydroxide	Sigma-Alderich CO	Cat#306568
Sucrose	Sigma-Aldrich Co.	Cat# S9378
Tricholine citrate	Sigma-Aldrich Co.	Cat# T0252
Sulforhodamine B	Sigma-Aldrich Co.	Cat# 230162
Brilliant blue FCF	Wako Pure Chemical Industry Ltd.	Cat# 027-12842
Paraformaldehyde	Electron Microscopy Sciences	Cat # 15710
Goat Serum, New Zealand origin	Gibco	Cat # 16210064

**Deposited data**		

All analyzed data	This paper	https://doi.org/10.6084/m9.figshare.25763694

**Experimental models: Organisms/strains**		

**Genotype**		
*Ir7a*^*1*^	Dr. Y. Lee	Rimal et al.^30^
*Ir7g*^*1*^:y^1^w^∗^Mi{y^+mDint2^=MIC}Ir7g^MI06687^	Bloomington *Drosophila* Stock Center	BDSC:42420
*Ir8a*^*1*^:w[∗]TI{w[+m∗]=TI}Ir8a[1];Bl[1]L[2]/CyO	Bloomington *Drosophila* Stock Center	BDSC:23842
*Ir10a*^*1*^:*w*^*1118*^Mi{GFP^E.3xP3^=ET1}Ir10a^MB03273^	Bloomington *Drosophila* Stock Center	BDSC:41744
*Ir21a*^*1*^:*w*^*1118*^;PBac{w^+mC^=PB}Ir21a^c02720^	Bloomington *Drosophila* Stock Center	BDSC:*10975*
*Ir25a*^*2*^	Dr. L. Voshall	Benton et al.^33^
*Ir47a*^*1*^	Dr. Y. Lee	Hiroi et al.^1^
*Ir47a-GAL4*	Bloomington *Drosophila* Stock Center	BDSC 60695
*Ir48a*^*1*^:*w*^*1118*^;Mi{GFP^E.3xP3^=ET1}Ir48a^MB09217^	Bloomington *Drosophila* Stock Center	BDSC:26453
*Ir48b*^*1*^:*w*^*1118*^;Mi{GFP^E.3xP3^=ET1}Ir48b^MB02315^	Bloomington *Drosophila* Stock Center	BDSC:23473
*Ir51b*^*1*^:*w*^*1118*^;PBac{w^+mC^=PB}row^c00387^ Ir51b^c00387^	Bloomington *Drosophila* Stock Center	BDSC:10046
*Ir52a*^*1*^	Dr. Y. Lee	Rimal et al.^30^
*Ir52b*^*1*^:*w*^*1118*^;Mi{GFP^E.3xP3^=ET1}Ir52b^MB02231^/SM6a	Bloomington *Drosophila* Stock Center	BDSC:25212
*Ir52c*^*1*^:*w*^*1118*^;Mi{GFP^E.3xP3^=ET1}Ir52c^MB04402^	Bloomington *Drosophila* Stock Center	BDSC:24580
*Ir56a*^*1*^	Dr. Y. Lee	Rimal et al.^30^
*Ir56b*^*1*^:*w*^*1118*^;Mi{GFP^E.3xP3^=ET1}Ir56b^MB09950^	Bloomington *Drosophila* Stock Center	BDSC:27818
*Ir56d*^*1*^*:w[∗];Ir56d[1]*	Bloomington *Drosophila* Stock Center	BDSC:81249
*Ir60b*^*3*^	Dr. Y. Lee	Rimal et al.^30^
*Ir62a*^*1*^:y^1^w∗;Mi{y^+mDint2^=MIC}Ir62a^MI00895^Iml1^MI00895^/TM3, Sb^1^Ser^1^	Bloomington *Drosophila* Stock Center	BDSC:32713
*Ir67a*^*1*^:y^1^w∗;Mi{y^+mDint2^=MIC}Ir67a^MI11288^	Bloomington *Drosophila* Stock Center	BDSC:56583
*Ir75d*^*1*^:*w*^*1118*^;Mi{GFP^E.3xP3^=ET1}Ir75d^MB04616^	Bloomington *Drosophila* Stock Center	BDSC:*24205*
*Ir76b*^*1*^	Dr. C. Montell	Zhang et al.^8^
*Ir85a*^*1*^:*w*^*1118*^;Mi{GFP^E.3xP3^=ET1}Ir85a^MB04613^ Pif1A^MB04613^	Bloomington *Drosophila* Stock Center	BDSC:*24590*
*Ir92a*^*1*^:*w*^*1118*^;Mi{GFP^E.3xP3^=ET1}Ir92a^MB03705^	Bloomington *Drosophila* Stock Center	BDSC:*23638*
*Ir94a*^*1*^	Dr. Y. Lee	Rimal et al.^30^
*Ir94b*^*1*^:*w*^*111*8^;Mi{GFP^E.3xP3^=ET1}Ir94b^MB02190^	Bloomington *Drosophila* Stock Center	BDSC*:23424*
*Ir94c*^*1*^	Dr. Y. Lee	Rimal et al.^30^
*Ir94d*^*1*^:y^1^w[;Mi{y^+mDint2^=MIC}Ir94d^MI01659^CG17380^MI01659^	Bloomington *Drosophila* Stock Center	BDSC:33132
*Ir94f*^*1*^:y^1^w∗;Mi{y^+mDint2^=MIC}Ir94f^MI00928^	Bloomington *Drosophila* Stock Center	BDSC:33095
*Ir94g*^*1*^:w^1118^;Mi{GFP^E.3xP3^=ET1}Ir94g^MB07445^	Bloomington *Drosophila* Stock Center	BDSC:25551
*Ir94h*^*1*^	Dr. Y. Lee	Rimal et al.^30^
*Ir100a*^*1*^:*w*^*1118*^;P{w^+mC^=EP}Ir100a^G19846^CG42233^G19846^	Bloomington *Drosophila* Stock Center	BDSC:*31853*
*Ir20a* RNAi	Vienna *Drosophila* Resource Center	VDRC:8658
*Ir47a* RNAi	Vienna *Drosophila* Resource Center	VDRC:11812
*Ir51b* RNAi	Vienna *Drosophila* Resource Center	VDRC:29984
*Ir52a* RNAi	Vienna *Drosophila* Resource Center	VDRC:37173
*Ir92a* RNAi	Bloomington *Drosophila* Stock Center	BDSC:58205
*Ir94f* RNAi	Vienna *Drosophila* Resource Center	VDRC:109702
*elav*-*GAL4*;*UAS-Dicer2*	Bloomington *Drosophila* Stock Center	BDSC:25750
*UAS*-*Ir51b*	Dr. Y. Lee	Dhakal et al.^42^
*Gr33a*^*1*^	Dr. C. Montell	Moon et al.^26^
*Gr33a*-*GAL4*	Dr. C. Montell	Moon et al.^26^
*ΔGr32a*	Dr. H. Amrein	Miyamoto and Amrein^53^
*Gr66a*^*ex83*^	Dr. C. Montell	Moon et al.^54^
*Gr89a*^*1*^	Korea *Drosophila* Resource Center (KDRC)	Sung et al.^55^
*Ir7c*^*GAL4*^	Dr. M. Gordon	McDowell et al.^15^
*Ir20a*^*1*^	Dr. A. Dahanukar	Ganguly et al.^39^
*ppk23*-*GAL4*	Dr. K. Scott	Thistle et al.^56^
*ppk28*-*GAL4*	Dr. H. Amrein	Cameron et al.^28^
*Gr64f*-*GAL4*	Dr. A. Dahanukar	Dahanukar et al.^57^
*UAS*-*Kir2.1*	Bloomington *Drosophila* Stock Center	BDSC*:6595*
*UAS-hid*:*P{w[+mC]=UAS*-*hid.Z}2*/*CyO*	Bloomington *Drosophila* Stock Center	BDSC: 65403
*UAS-Ir52a*	This paper	N/A
*UAS-Ir94f*	Dr. Y. Lee	Pradhan et al.^43^
*BC*/*CyO*;*Gr66a*-*I*-*GFP*,*UAS*-*Dsred*/*TM6b*	Dr. J.R. Carlson	Weiss et al.^44^
*UAS*-*mCD8*::*GFP*/*CyO*	Bloomington *Drosophila* Stock Center	BDSC:5137
*OtopLa*^*1*^	Bloomington *Drosophila* Stock Center	Ganguly et al.^11^
*alka*	Bloomington *Drosophila* Stock Center	BDSC:56318
*alka*-*RNAi*	Bloomington *Drosophila* Stock Center	BDSC:26250

**Software and algorithms**		

Origin Pro Version	Dr. Y. Lee	https://www.originlab.com (Origin Lab Corporation, RRID: SCR 002815)
GraphPad Prism Version 8.0	Dr. Y. Lee	https://www.graphpad.com (RRID: SCR 002798)
Autospike 3.1 software	Dr. Y. Lee	https://www.syntech.co.za/

### Resource availability

#### Lead contact

Further information and requests for reagent should be directed to and will be fulfilled by the Lead contact, Youngseok Lee (ylee@kookmin.ac.kr).

#### Materials availability

This study did not generate new reagents.

#### Data and code availability

• This study did not generate any unique datasets or code.
• The raw data has been deposited at Figshare and is publicly accessible as of the date of publication. The DOI is listed in the key resources table.
• Any additional information required to analyze the data reported in this paper is available from the lead contact upon request.

### Experimental model and study participant details

All experiments were performed with adult male and female *Drosophila melanogaster*. Binary food choice assays and tip recordings were conducted using 3 to 7-day-old flies*.* The mutants and transgenic lines used in this study are listed in the key resources table.

### Method details

#### Binary food choice assay

In alignment with a prior investigation, we conducted binary food choice experiments.^58^ Within a humid chamber, a group of 50 to 70 flies (3–6 days old, both sexes) were subjected to an 18-hour starvation period. The subsequent steps involved the preparation of two distinct food sources, both containing 1% agarose. The initial food source was supplemented with 2 mM of sucrose, while the second source contained different concentrations of NaOH or KOH with 2 mM sucrose. To differentiate between the two food sources, blue food coloring dye (0.125 mg/mL brilliant blue FCF) was added to one, and red food coloring dye (0.1 mg/mL sulforhodamine B) was added to the other. The prepared solutions were alternatively distributed into wells of a 72-well microtiter dish (cat. no. 438733; Thermo Fisher Scientific, Waltham, MA, USA). Within roughly 30 minutes of preparing the food, approximately 50 to 70 starved flies were introduced to the plate in each trial (n). The flies were allowed to feed for 90 minutes at room temperature by incubating the dishes within a dark, humid container. Subsequently, the tested flies were carefully frozen at a temperature of −20°C. Their abdominal coloration was then observed and categorized using a stereomicroscope. The classification involved categorizing flies as blue (N_B_), red (N_R_), or purple (N_P_) based on visual assessment of the abdomen color. Each fly was assigned a value depending on the color it displayed after consuming exclusively or a combination of food sources (red, blue, or purple). For each fly, the preference index (PI) was computed, a value derived from the dye and tastant combinations. The formula used for this calculation was (N_R_ – N_B_) / (N_R_ + N_B_ + N_P_) or (N_B_ – N_R_) / (N_R_ + N_B_ + N_P_). A preference index of either 1.0 or -1.0 denoted a significant inclination toward one of the food alternatives. Meanwhile, a PI of 0.0 indicated no bias among the flies towards either option.

#### Tip recording assay

The process of electrophysiology, specifically the tip recording assay, was executed in accordance with previously outlined procedures.^59^ Flies of both sexes, aged between 4–7 days, were gently anesthetized on ice. Next, a reference glass electrode, containing Ringer's solution, was delicately introduced into the thorax of the flies. This electrode was then incrementally advanced towards the proboscis of the fly. This procedure was replicated across various days to ensure robustness. To activate the sensilla, a recording pipette (with a tip diameter of 10 to 20 μm) was attached to a preamplifier. This pipette was filled with a mixture of chemical stimulants in a 30 mM tricholine citrate (TCC) solution, which served as the electrolyte solution. Employing a Syntech signal connection interface box and a band-pass filter spanning 100 to 3000 Hz, the signals obtained from the recordings were amplified by a factor of 10. Action potentials were then captured at a sampling rate of 12 kHz and subsequently analyzed using Autospike 3.1 (Syntech, Hilversum, The Netherlands). To ensure signal integrity, all recordings were conducted at intervals of 1 minute. This approach guaranteed the acquisition of accurate and reliable signals.

#### Proboscis extension response (PER) assay

The method for conducting the proboscis extension response (PER) experiment was replicated from a previous study.^60^ In this experiment, approximately 20 to 25 flies of both sexes, aged between 3 to 7 days, were subjected to a period of starvation lasting 18 to 20 hours. During this time, the flies were kept in vials containing 1% agarose. After a brief period of anesthetization on ice, the flies were gently placed within a narrow 20–200 μL pipette tip, ensuring that they were not harmed. The tip of the pipette was then carefully trimmed using a paper cutter blade to expose the fly's head. By doing so, the head and proboscis were made accessible outside the pipette tip, allowing for the application of stimuli to the proboscis. To eliminate any potential biases stemming from thirst, the flies were initially given access to water. Subsequently, a 2% sucrose solution was employed for both the initial stimulation and as a positive control. The actual stimuli, which were substances to be tasted, were introduced using Kimwipe paper as the delivery medium. Next, the proboscis was exposed to dampened pieces of Kimwipe paper soaked in either a 2% sucrose solution or 2% sucrose with different NaOH concentrations. Flies that did not exhibit a response to the sucrose solution during the first exposure were excluded from the experiment. The same conditions that were used during the initial exposures were replicated for the subsequent exposures. In each round of testing, more than 10 flies were subjected to the experiment. This entire process was repeated a total of six times to ensure reliability and consistency of results.

#### Immunohistochemistry

This procedure closely followed the protocol outlined by a previous paper.^5^ The labella of the flies were dissected and fixed with 4% paraformaldehyde (Cat#15710; Electron Microscopy Sciences, Hatfield, PA, USA) in 1X phosphate-buffered saline and 0.2% Triton X-100 (PBS-T) for 15 minutes at room temperature. After fixation, the tissues underwent three careful washes with PBS-T and were subsequently bisected with a razor blade. Following this, the samples were incubated in blocking buffer (0.5% goat serum in 1X PBST) for 30 minutes at room temperature. Primary antibodies (1:1000 dilution; mouse anti-GFP [Cat# A11120; Molecular Probes, Eugene, OR, USA], rabbit anti-DsRed [Cat #632496; TaKaRa Bio, San Jose, CA, USA]) were added to freshly prepared blocking buffer and allowed to incubate with the samples overnight at 4°C. The samples were then washed three times with PBS-T at 4°C and exposed to secondary antibodies (1:200 dilution in the blocking buffer; goat anti-mouse Alexa Fluor 488 [Cat# A11029; Thermo Fisher], goat anti-rabbit Alexa Fluor 568 [Cat# A11011, Thermo Fisher/Invitrogen]) for 4 hours at 4°C. Afterward, the tissues underwent three additional washes with PBS-T before being mounted in 1.25x PDA mounting buffer (37.5% glycerol, 187.5 mM NaCl, and 62.5 mM Tris, pH 8.8) and examined using a confocal microscope.

#### RT-PCR analyses

Labellum samples were isolated from adult flies of control (*w*^*1118*^) or bitter-sensing neuron (*Gr33a-GAL4*)-ablated flies using *UAS-hid*. The total RNA extraction was performed using TRIzol (Invitrogen, Carlsbad, CA, USA), followed by cDNA synthesis using the Avian Myeloblastosis Virus (AMV) reverse transcriptase (Promega, Madison, WI, USA). To perform RT-PCR we used the *Ir20a* primers : 5' - TGG CAA GCT TGA ACC GTA-3' and 5'- GTA CAA CAG CTC CAT TAT G-3', *Ir51b* primers: 5′-GGC GCT AAC AAA CGC TGC TTAC -3′ and 5′-CAG AGC TGA CAG TAT CCA ACC AA-3’, *Ir52a* primers: 5'-ATG GCT CTT GGG TGG TCA GT-3' and 5'-TTA CCA TCT ACA CCT CCA TA-3', *Ir92a* primers: 5'-GCC AAT TGC TTC GGA TTA TAG -3' and 5'-CGT GCA AAA CAA TGC GAT TT- 3', and *Ir94f* primers: 5'-ATG TGG CAA CAA GTG CTG TT-3' and 5'-TAG TGA ACA GTG AGC TCG AT -3'. The *tubulin* primers were 5′-TCC TTG TCG CGT GTG AAA CA-3′ and 5'-CCG AAC GAG TGG AAG ATG AG-3'. The Lamp Taq DNA polymerase (BioFact™ ) was used. The RT-PCR reactions underwent 35 cycles to amplify the fragments of the target gene. The initial denaturation was for 5 minutes at 95°C, denaturation was for 30 seconds at 95°C, initial extension was for 30 seconds at 72°C, and extension was for 2 minutes at 72°C. The annealing temperature for *Ir20a*, *Ir51b*, *Ir52a*, *Ir92a*, and *Ir94f* was 54°C, 61°C, 54°C, 58°C, and 54°C, respectively, for 1 minute. To get more precise amplification, the annealing temperature was adjusted by increasing < 2.5°C to the calculated value of melting temperature (T_m_) provided by the primer manufacturer, COSMO_GENETECH_, Korea. Subsequently, the resulting RT-PCR products were analyzed to assess gene expression levels. To verify the integrity and purity of the RNA preparation and to ensure the absence of genomic DNA contamination, a No Reverse Transcriptase (no-RT) sample was included. This control involved RNA that did not undergo treatment with AMV reverse transcriptase enzymes before proceeding to PCR.

### Quantification and statistical analysis

Data were plotted and analyzed using GraphPad Prism version 8.0 (RRID: SCR 002798). All experiments were repeated on separate days. The number of trials for each experiment is represented by dots in the graphs. The standard error of the mean (SEM) is represented by error bars. Using single-factor analysis of variance (ANOVA) and Scheffe's post hoc analysis, multiple datasets were compared. OriginLab (Northampton, MA, USA; RRID: SCR 002815) was used for all statistical analyses.

Data information: All error bars represent the standard error of the mean (SEM). Single-factor analysis of variance (ANOVA), coupled with Scheffe’s post hoc test, was conducted to compare multiple datasets. Asterisks indicate statistical significance compared to the control. ∗∗p < 0.01. This is applicable for Figures 1B–1E, 2A, 2C–2F, 3A–3D, 4A–4C, 4E, 4F, S1, and S2.
