# Supplementary material for: Avoiding alkaline taste through ionotropic receptors
Source: iScience. 2024 May 23;27(6):110087. doi: 10.1016/j.isci.2024.110087 (PMC11214294; doi:10.1016/j.isci.2024.110087)
Supplement: Document S1. Figures S1–S3 [file mmc1.pdf]

iScience, Volume *27*

Supplemental information

Avoiding alkaline taste through ionotropic receptors

Prakash Pandey, Bhanu Shrestha, and Youngseok Lee

Supplemental Information

Avoiding Alkaline through Ionotropic Receptors

Prakash Pandey, Bhanu Shrestha, and Youngseok Lee

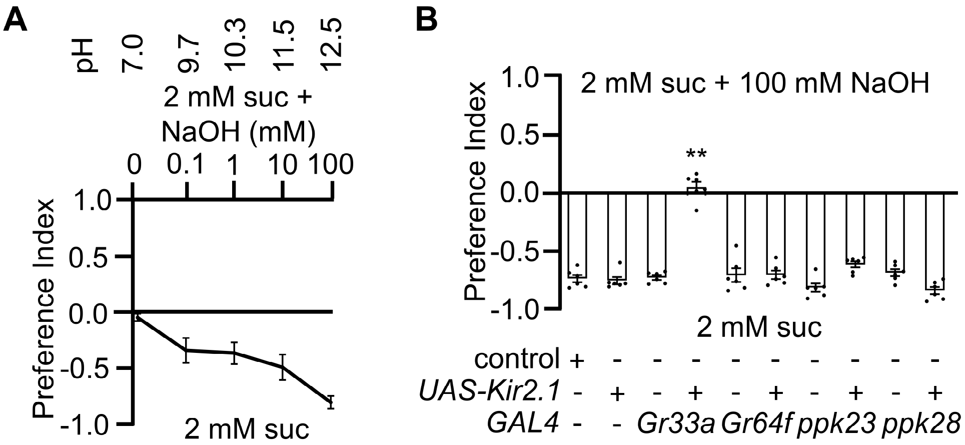

Figure S1 (related to Figure 1). Binary food choice response of control flies in different concentrations of NaOH and specific GRN-ablated flies with 100 mM NaOH
(A) A dose-dependent binary food choice assay of control (*w[1118]*) flies using NaOH in the range of 0-100 mM (n = 6). **(B)** Feeding assays of specific GRN-ablated flies using 100 mM NaOH. +/- indicates the presence or absence of the transgene, respectively (n = 6). Data information: All error bars represent SEMs. Single-factor ANOVA, coupled with Scheffe's post hoc test, was conducted to compare multiple datasets. Asterisks indicate statistical significance compared to control. $^{**}p < 0.01$.

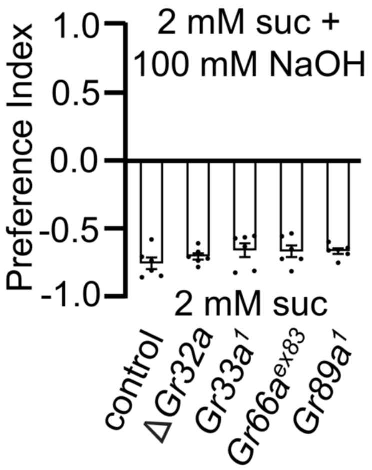

Figure S2 (related to Figure 3). Binary food choice response of specific bitter sensing GRs with 100 mM NaOH

Binary food choice assays of control, Δ*Gr32a*, *Gr33a^{1}*, *Gr66a^{ex83}*, and *Gr89a^{1}* flies in the presence of 100 mM NaOH (n = 6). Data information: All error bars represent SEMs. Single-factor ANOVA, coupled with Scheffe's post hoc test, was conducted to compare multiple datasets.

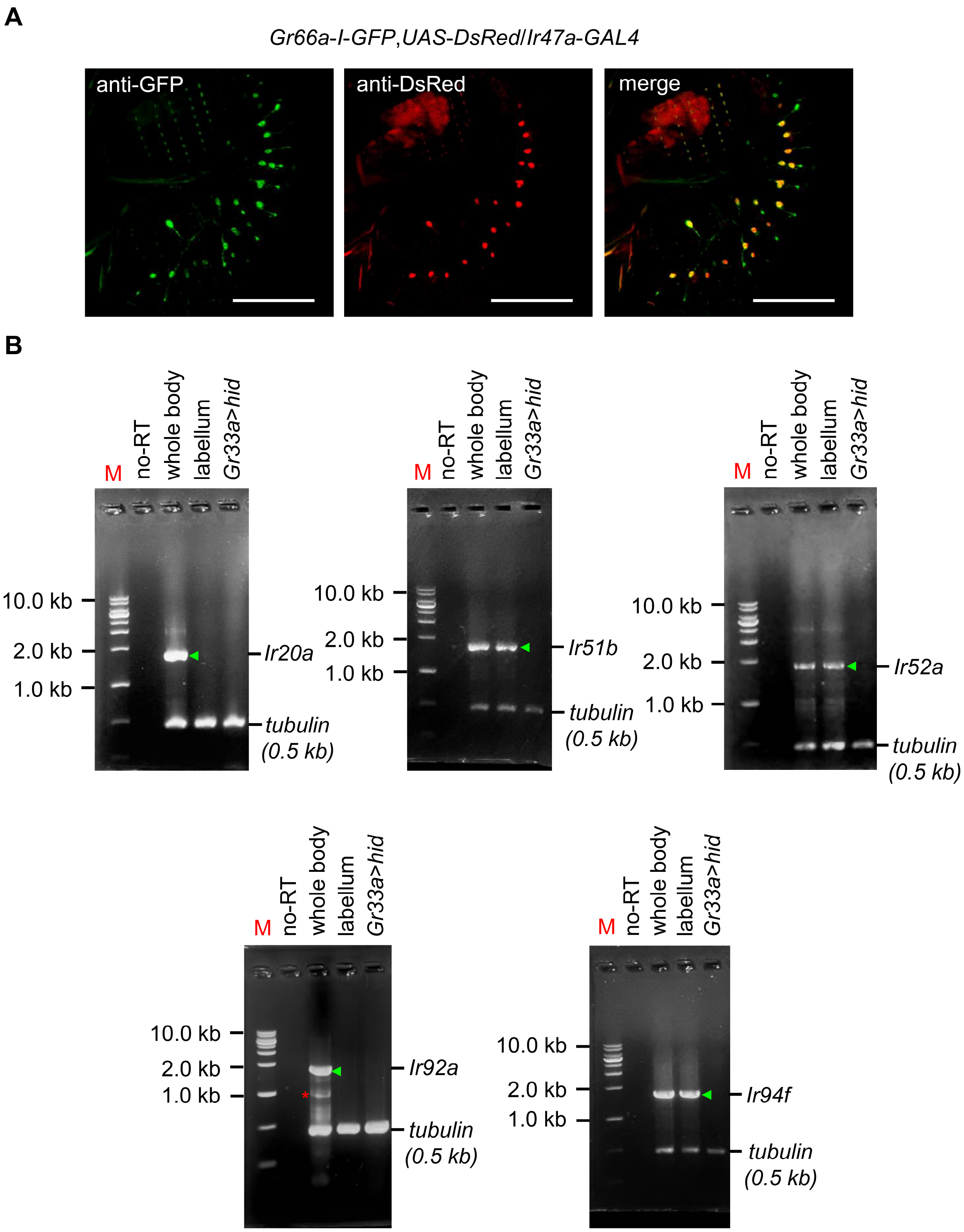
A
Gr66a-I-GFP,UAS-DsRed/Ir47a-GAL4
anti-GFP
anti-DsRed
merge
B
M
no-RT
whole body
labellum
Gr33a>hid
10.0 kb
2.0 kb
1.0 kb
Ir20a
tubulin
(0.5 kb)
Ir51b
Ir52a
Ir92a
Ir94f

Figure S3 (related to Figure 4). Expression of the *Ir47a-GAL4* reporter in subsets of *Gr66a*$^{+}$ GRNs and RT-PCR analysis of the *Irs* in whole body, labellum, and bitter GRN

(A) *Gr66a-I-GFP* labeled all bitter responsive GRNs (anti-GFP, green), *UAS-DsRed* was expressed under the control of the *Ir47a-GAL4* (anti-DsRed, red). Most but not all Ir47a-positive cells were detected in a single confocal image. The merged image revealed a higher number of co-labeled neurons. The scale bars represent 50 µm. **(B)** A gel picture of RT-PCR results showing the expression of *Ir51b*, *Ir52a*, and *Ir94f* in the labellum. The respective genes were amplified using RT-PCR in the control (w^{1118}) and *Gr33a-GAL4*/*UAS-hid* (bitter-sensing GRNs ablated) flies. Amplified tubulin products (0.50 kb) were used as the control for PCR reaction. “M” indicates the DNA ladder marker. The green arrowhead represents the expected band size, and the red asterisk demonstrates the unspecific band. Band sizes of *Ir20a*, *Ir51b*, *Ir52a*, *Ir92a*, and *Ir94f* are 1.65 kb, 1.70 kb, 1.80 kb, 1.79 kb, and 1.64 kb, respectively.
